# Fluorescence Monitoring Oxidation of Extra Virgin Olive Oil Packed in Different Containers

**DOI:** 10.3390/molecules27217254

**Published:** 2022-10-26

**Authors:** Elísabet Martín-Tornero, Antonio Fernández, Isabel Durán-Merás, Daniel Martín-Vertedor

**Affiliations:** 1 Departamento de Química Analítica, Universidad de Extremadura, 06006 Badajoz, Spain; 2 Technological Institute of Food and Agriculture, CICYTEX-INTAEX, Junta de Extremadura, Avda. Adolfo Suárez s/n, 06007 Badajoz, Spain

**Keywords:** olive oil, fluorescence spectroscopy, olive oil conservation, packaging, quality parameters

## Abstract

‘Picual’ olive oil was stored in different types of containers for 10 months and monitored via quality parameters. In combination with the mentioned analysis, non-destructive fluorescence spectroscopy was performed combined with multivariate analysis to monitor and quantify oil quality levels. Excitation emission matrices (EMMs) were analyzed using parallel factor analysis (PARAFAC). According to the quality parameters, it was observed that Transparent Crystal (TC) and Opaque Crystal (OC) samples were the ones that deteriorated faster due to their higher exposure to light in comparison with Plastic (P) and Canned (C) samples. In a fast and non-destructive manner, the fluorescence spectroscopy-based prototype successfully monitored the oxidation changes in the EVOOs. Unfolded partial least squares (U-PLS) was used to generate a regression model to quantify quality parameters. Good correlation coefficients were found for the peroxide index, K_232_ and the oxidative stability index (r^2^ between 0.90 and 0.94 for cross-validation and validation). For all of that, the results obtained confirmed the ability of fluorescence spectroscopy to monitor the quality of olive oil and EEMs combined with U-PLS can be used to analyze these parameters, eluding the classical methods.

## 1. Introduction

Due to its healthy nutritional properties, it is well known that olive oil is one of the most important products in the Mediterranean diet. Producing countries like Spain or Italy take advantage of this producer role in the sector’s economy by ensuring its quality with exhaustive controls, a functional elaboration process, olive oil categorizations (Olive oil, OO; virgin olive oil, VOO; and extra virgin olive oil, EVOO) and packaging. These initiatives help to maintain beneficial substances like unsaturated fatty acids, polyphenols, vitamin E, carotenoids, sterols, etc. At the present time, dietary care and healthy alimentary habits, like consuming EVOO, are growing among consumers, especially when it comes to antioxidant intake due to its long and short terms benefits [1,2] such as preventing and reducing certain gastrointestinal diseases [3].

Having that in mind, the concentration of these bioactive compounds is not only influenced by the cultivar agronomic conditions (such as the cultivar type, maturation stage, agroclimatic conditions and agronomical practices), raw material, harvesting, fruit storage and extraction technology, but also by each factor which can affect it during its commercial life [4,5,6]. Therefore, some studies have proven that oxygen, light and temperature are variables responsible for increasing deteriorative processes in EVOO as a consequence of oxidative and hydrolytic reactions [7]. When controlling these parameters, EVOO shelf-life can be between 12 and 18 months [8], reaching the second year when good storage is achieved together with well-sealed packaging [9]. Several articles have been published where the influence of the storage time on quality was evaluated [10].

However, EVOO quality and shelf-life is reduced by oxidation, which represents one of the greatest EVOO quality degradation factors during storage [11,12] and which can be counteracted by the antioxidant activity of polyphenolic compounds and tocopherols. The most susceptible fraction to oxidation is the lipid one, which produces an increase of carbonyl and aldehyde compounds that prompt off-flavors and ‘oxidative rancidity’ at the end, being unsuitable for human consumption [13].

Auto-oxidation contributes to the degradative processes of the olive oil, happening even with lack of light. This is due to a free radical mechanism where, at the beginning, the absorption of O_2_ results in the formation of hydroperoxides. On the other hand, when EVOO is exposed to light, photo-oxidation occurs through the action of natural photosensitizers such as chlorophyll. As an outcome, storage and packing conditions of EVOO become crucially important [5,14]. Stefanoudaki et al. [10] studied the evolution of VOO during 15 months of storage inside and outside warehouse conditions, but they did not maintain a controlled temperature.

In order to avoid oxidation by different sources, dark containers, optimal containers that can avoid oxygen penetration, and low temperatures during storage can be helpful in preserving EVOO quality [12,14,15]. Previous studies have evaluated different packaging materials, light transmission influence, temperature effects and time of storage concerning quality physicochemical parameters (such as acidity, peroxide index, K_232_ and K_270_), sensory attributes and shelf-life of EVOO, finding how organoleptic and quality properties are lost as time goes by [12,15,16].

Fluorescence spectroscopy has been proposed as an alternative technique to analyze and monitor olives and olive oil. It has the advantages over the other conventional methods of its speed of analysis and the minimum sample preparation with the absence of solvents and reagents. Excitation emission matrices (EEMs) have been applied with the aim of determining phenolic compounds in olives [17] or of discriminating between and classifying virgin, pure and olive pomace oil [18], olive oils coming from different regions [19] or olive oils coming from two varieties and submitted to two different irrigation treatments [4]. Moreover, fluorescence spectroscopy has been proposed to monitor olive oil during storage in different conditions. In this sense, the influence of factors such as UV irradiation, sunlight exposure and temperature up to 80 °C were studied [20]. Mishra et al. [21] monitored oils from three different olive varieties exposed and not exposed to light and Lobo Prieto et al. [22] studied the changes in the quality parameters and the relationship with the EEMs of four different cultivars. Fluorescence spectroscopy was also used to monitor oxidation level of four edible vegetable oils during storage at 60 °C [23]. The characteristics of some types of containers were also shown to influence the quality parameters of olive oil, as explained above, and the influence of storage in clear and green glass bottles exposed to light and in darkness was monitored using fluorescence spectroscopy [24]. However, the use of other different commonly used containers has not been studied to date by fluorescence. The aim of this study was to monitor the evolution of the fluorescence fingerprint of the virgin olive oil, during storage at room temperature, in four common different containers. At the same time, excitation emission fluorescence matrices in combination with chemometric algorithms was proposed as an alternative to conventional methods to determine the physicochemical and quality parameters of the olive oil.

## 2. Results and Discussion

### 2.1. Influence of Packaging on the Quality Parameters during Storage at Room Temperature

**Figure 1:**
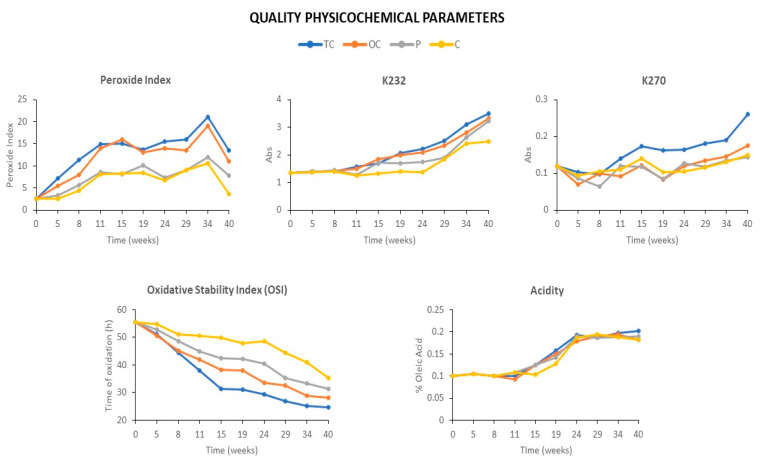
Evolution of quality parameters of olive oil with the storage time in different containers.

**Figure 2:**
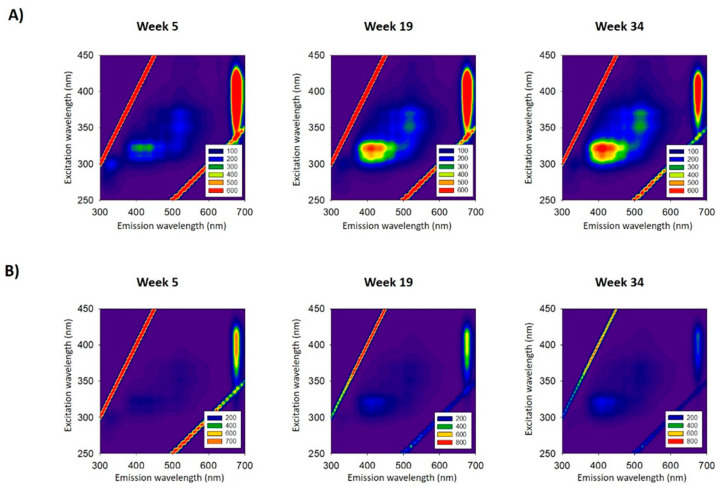
Contour plot of the EEMs of the samples stored in transparent crystal at three different storage times. (**A**) 700 V and (**B**) 630 V.

Quality indexes were performed for the EVOO samples stored in different containers for 40 weeks (Figure 1). An increase in peroxide index during the first eleven weeks of the study can be observed, followed by a period with values practically constant from week 15 to 29. Around week 34 a slight increase is detected, and a drastic decrease at week 40. In Figure 2A, two clearly different groups can be identified. In the first one, with crystal containers, it can be detected that independently of the crystal nature (TC and OC), the values of peroxide indexes are higher along the studied period, surpassing the EVOO legal limit at week 34. The second is composed of non-crystal containers (Pl and C) and in this, the values of the peroxide index are lower, which indicates that samples conserved in these containers were less affected by the oxidation. This observation can be explained by how daylight strikes in the sample through the container and how the container protects the oil matrix from degradation. Samples in TC containers were the ones which presented the higher peroxide index, finding a maximum of 21.1 mEqO_2_∙kg^−1^. The transparency of the material and its lack of light covering cause different light frequencies to pass through the material, affecting the EVOO, and promoting oxidation and matrix degradation. Similar results were found by Dabbou et al. [24]. However, OC receptacles slightly shield the samples from light irradiation, not surpassing the extra virgin category threshold in any of the studies. With respect to plastic and can vessels, they were able to filter light with such efficiency that the peroxide index did not become higher than 12.0 mEqO_2_∙kg^−1^. Other researchers observed maximum values of the peroxide index from month 9 [25]. In addition, Alvarruiz et al. [26] underlined that long-time storage of EVOO increases its peroxide concentration, observing a significant drop after its maximum as in the present research.

Some types of plastic do not correctly protect olive oil from oxidation, letting light and oxygen pass through the material and triggering the oxidation mechanism. It was observed that the peroxide index increases during a 12 month storage period [25,27]. Significant differences were also found by Sanmartin et al. [5] in containers like tinplate, with an increase of peroxide values of 77% after the storage period.

Regarding acidity, initially the percentage of acidity is 0.1% and remains practically constant during the first 11 weeks (Figure 1). From week 15 to 24, an increase up to 0.2% is observed in all packages, keeping this value practically constant until week 40. From week 15 to 24, olive samples in C containers present the lowest values of % oleic acid. The opaque character of metallic cans prevents light penetration through the material and its corresponding effect in EVOO. From week 24 to the end of the study, there are no significant differences between the containers used until week 40. It can be said that samples conserved in TC containers can be differentiated from the rest and also they presented the highest acidity. However, all the containers studied to store the EVOO are effective so that the oil remains in the extra virgin olive oil category (EU Regulation 2568/1991 modified). Previous researchers have found the same trend [26,27].

Data reveal that K_232_, which is the corresponding parameter to evaluate primary oil oxidation, shows a similar behavior in terms of the percentage of acidity (Figure 2). It remains constant during the first 11 weeks in all the containers studied and increases progressively from week 11 to 40 (Figure 1). From week 19 onwards, differences between containers started to become apparent, reaching a maximum value of 3.5 for oil samples conserved in TC containers. Oil samples in can containers present the lowest values with regards to this parameter in the study, reaching values below the set limit (≤2.5) on week 40. This is an interesting observation because these containers are able to increase the EVOO shelf-life by at least 2.5 months. Results concerning K_270_ did not show clear behavior until week 19. From that week on, K_270_ increased, reaching its highest value in week 40 (Figure 1). EVOO resisted secondary oxidation thanks to the container’s light tolerance, with Pl and C being the receptacles that better protected olive oil samples. Olive oils in TC containers show the highest values, exceeding the set limit for the EVOO category (≤0.22). In PT and C packaging, the evolution of this parameter is softer, showing a slight increase up to week 40 with maximum values of 0.15. Similar results were found in other studies [26,27]. In general, glass containers are preferable to plastic vessels for EVOO packaging in order to avoid oxygen permeation [25].

Oxidative stability index (OSI) data showed that, for all packaging, a decrease is observed in the study (Figure 1). During the first 15 weeks of the trial, a considerable decrease in this parameter was observed, from 59 to 50 h for C containers and from 59 to 30 h for TC containers. From that point on, the decrease slows down until week 40. The least significant changes were found with type C containers with a reduction in the OSI of 36%. However, the most significant reduction—of 55.5%—was found with TC containers. Alvarruiz et al. [26] also found a stability loss in the ‘Picual’ variety of 38% after a long-time storage. A similar tendency was indicated by Iqdiam et al. [27] in different cultivars and oxygen concentrations.

As was expected, oil samples in TC containers were the samples that suffered the deepest oxidation process. In contrast, the C container was the most effective in terms of preserving chemical characteristics to prevent EVOO oxidation due to degradation from exposure to light. This could be justified due to the phenolic compound content. Metallic materials did not let light affect the olive oil samples, preventing phenol degradation and, therefore, maintaining antioxidant resistance. In previous studies, other researchers confirmed the hypothesis that olive oil stability is primarily related to the level of unsaturated fatty acids and antioxidant molecules such as phenols that have a linear correlation with OSI in EVOO [25,27,28,29].

### 2.2. Fluorescence Monitoring

#### 2.2.1. EEM Description

In order to obtain the fluorescence fingerprints from the different oil samples packed in different containers, EEMs were registered at the same time that the quality parameters were determined. The conditions employed to collect the EEMs are described in Section 3.6. Figure 2 shows the EEMs from EVOO samples packaged in transparent crystal containers at three different storage times and recorded at two different voltages (630 and 700 V) of the photomultiplier tube. In general terms, the EEMs exhibited physiognomy similar to previous reports [4,22].

In Figure 2, four different fluorescence regions can be observed. In accordance with the literature, the emission spectral region between 320 and 350 nm (excitation between 280 and 310 nm) corresponds to polyphenols and tocopherols present in the olive oil [17,22]. As can be seen, the fluorescence of this region decreases with the storage time, and this behavior was similar for all tested containers. As stated in the bibliography, the fluorescence of this area decreases with storage time due to the action of light, which has been corroborated in the different containers used.

The opposite occurs with the other two more representative spectral regions, one of them with an emission range of 380–460 nm and an excitation range between 320 and 340 nm, and the other with an emission range of 480–550 nm and an excitation range of 350–390 nm, whose fluorescence intensities increase with storage time. These spectral regions have been associated with degradation processes [30].

Moreover, these regions have been related to the primary and secondary oxidation products, respectively. These primary and secondary oxidation products often result from the auto-oxidation and photo-oxidation of oils occurring during the storage, in which triplet oxygen (^3^O_2_) and singlet oxygen (^1^O_2_) react with the oil, respectively [21]. Although several factors can be involved in the formation of these oxidation products, in this study, and given that the only variable is the type of container, our goal is to relate the fluorescence fingerprint with these variables. Finally, the fourth spectral region with emission wavelengths higher than 600 nm, corresponding to chlorophylls and their derivatives, has been widely described in the literature [20,22]. As can be seen in Figure 2, the fluorescence decreases with storage time, as occurs in the first region.

#### 2.2.2. Application of PARAFAC to EEMs in the Different Containers

Since the evolution of the fluorescence of the regions corresponding to polyphenols and chlorophylls has been extensively studied [20,22,24], we have focused the study on the spectral region of the oxidation products, with the objective of figuring out the evolution of the fluorescence fingerprint in this region when different containers for olive oil samples are used. Therefore, the spectral region with excitation wavelengths between 295 and 375 nm and emission wavelengths between 384 and 580 nm was used in the study. To resolve the fluorescence profiles, multivariate exploratory algorithms, such as PARAFAC, were used and two different analyses were carried out, one with all the samples, independently of the container, and the second with the samples of each container.

In the first study, the EEMs were arranged in a three-dimensional structure with dimensions of 17 × 99 × 48 (excitation × emission × samples) and then decomposed with PARAFAC. PARAFAC was performed with one, two, three and four components in orderto select the optimal number. In all the cases, non-negative constraints were applied to all modes, given that all concentration and spectral values are always positive.

Two components were selected as the optimal number, taking into account the core consistency diagnostic (CORCONDIA) [31], the residual analysis [32] and the physiognomy of the loading.

**Figure 3:**
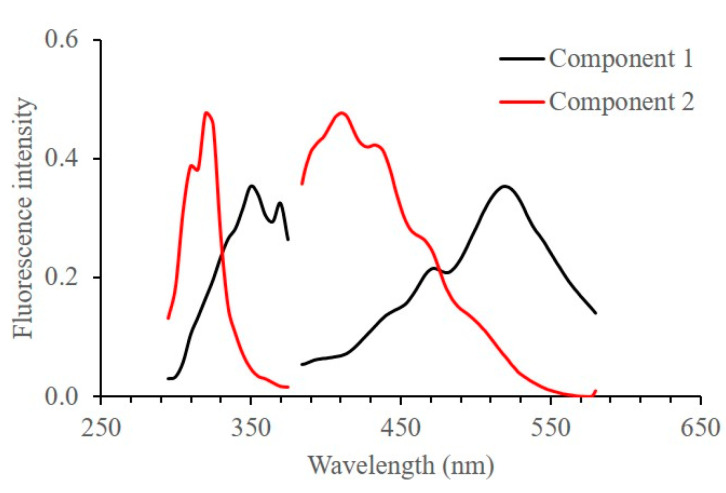
Excitation and emission PARAFAC loadings obtained with the complete set of samples.

Figure 3 shows the excitation and emission PARAFAC loadings of the two principal components. The excitation profile of the first component shows two maxima at 350 and 370 nm, and the emission profile presents a wide band with a maximum at 525 nm. The presence of fluorescent components with these wavelengths has already been previously described [33,34,35,36]. The assignment of this component has been widely discussed by several authors, ruling out that it can be assigned to vitamin E. The most likely assignment is the one proposed by Sikorska et al. [24], who consider that the fluorescent compounds responsible for this signal are formed during the oil storage. In addition, as indicated by Botosoa and Karoui [23], the width of its emission profile and its physiognomy, which is similar to that of a large number of compounds with short excitation wavelengths, indicate that this component corresponds to more than one analyte.

With respect to the second component, the excitation profile presents a maximum at 320 nm and a shoulder at 310 nm, and the emission profile has a maximum placed at 414 nm and two shoulders at 434 and 470 nm. This component has also been found in previous studies and it has been ascribed to primary and secondary oxidation products [4,22,33,37]. The same PARAFAC decomposition was performed independently with the samples of each container (12 samples for each type), obtaining the same number of components and similar loading profiles for all of them as when the complete data set was used.

#### 2.2.3. Fluorescence Evolution with the Storage Time in Different Containers

**Figure 4:**
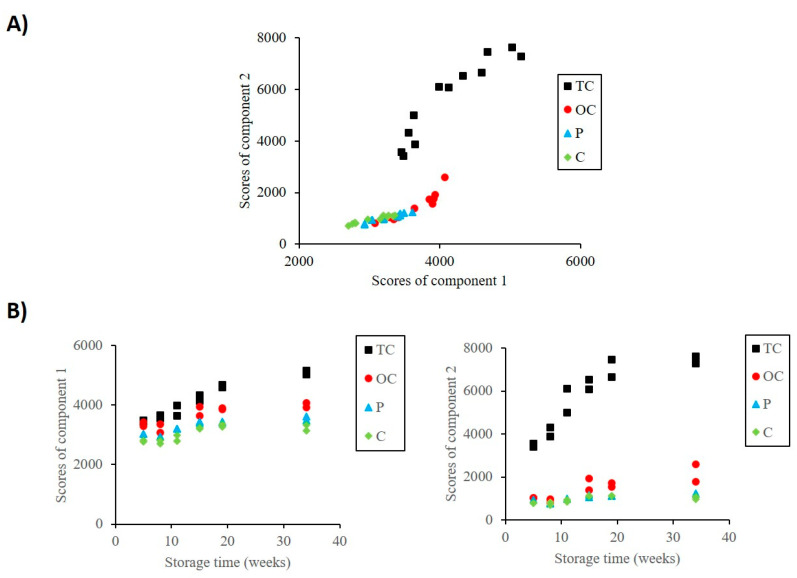
(**A**) PARAFAC scores of all the samples in different containers throughout the entire storage time. (**B**) Evolution of scores 1 and 2 across storage time.

With the aim of studying the evolution of the fluorescence fingerprint of the EVOO in each container type, the evolution of the PARAFAC scores of the two principal components in the spectral region selected were studied. Score values for both components over the 40 weeks for each container are plotted against each other in order to distinguish among the container types (Figure 4).

As can be seen, the score values for the samples in CT are very different from the samples stored in CO, Pl and C. Those samples present higher values with regard to the first and second component than the other containers and they can be easily distinguished. This suggests that the oxidation in those samples was faster than in the other samples due to the photo-oxidation, as was described above. Moreover, it can also be observed that the CO samples stored longer can also be distinguished from samples packed in CO and C containers because they have higher scores with regard to components 1 and 2. However, samples in Pl and C containers are not different if we consider these two components.

Moreover, it can be seen that all the containers show a sequential shift in the samples measured every 4 weeks along the component 1 and 2 axis. For a better visualization, the PARAFAC score values for the first and second components were plotted as a function of the storage time (Figure 4). The same tendency is observed for both components and all the containers. Score values increased as storage progressed until week 20, and, after that, they remained almost constant. It can also be appreciated that CT samples are those which present higher values for both components, followed by CO, Pl and C containers. CT samples present much higher values for component 2 scores, suggesting that the oxidation that has occurred in these samples is much greater than in the other containers. These results are in agreement with those obtained in the quality parameters, which were described above, and verify the ability of fluorescence spectroscopy to monitor the changes in virgin olive oil during storage.

### 2.3. Quantification of Quality Parameters by Using the EEMs

After the qualitative analysis of the evolution of the fluorescence of the olive oil across storage time in different containers, the relationship between EEMs and the quality parameters was evaluated. The physico-chemical data indicated the presence of oxidation products, so it is logical to investigate whether it is possible to quantify them by means of the fluorescent region selected in the previous studies.

U-PLS regression was selected to develop regression models between the quality parameters and the EEMs. Samples were divided into two data sets: the calibration set, with 70% of the samples used to optimize and build the model, and the validation set, with the remaining 30% of the samples, that will be used to validate the previous model. First, it was necessary to select the optimum number of components. This was achieved by using the calibration set and the Haaland and Thomas criterion [38]. The optimal number of components is given when the PRESS value is statistically not different to the minimum PRESS value. Four latent variables were selected in all the models. After a model was built, it was validated using the other samples. The results obtained for the different quality parameters (peroxide index, K_232_, K_270_ and OSI) in the cross-validation and the validation model are summarised in Table 1. The correlation coefficients show that the results obtained with U-PLS and the conventional methods are highly correlated for peroxide index, K_232_ and OSI (r^2^ higher than 0.90 in cross-validation and validation). For K_270_, good results were also found with a correlation coefficient higher than 0.80. The statistical parameters were evaluated through the root mean square error of cross-validation (RMSECV) and prediction (RMSEP), and the relative error of prediction (% REP). These values were considered low for peroxide index, K_232_, K_270_ and OSI.

The obtained results prove the accuracy and precision of the developed model for the physico-chemical parameters studied, and suggest that the EEMs combined with U-PLS are a good method to determine these parameters, avoiding the traditional methods.

## 3. Materials and Methods

### 3.1. Chemical Reagents

For the analysis of the physico-chemical regulated parameters, ethanol, diethyl ether, sodium thiosulphate, potassium iodine, phenolphthalein, starch, acetic acid, chloroform and cyclohexane, all of analytical grade, were purchased from Fisher Scientific (Fisher Scientific, Waltham, Massachusetts, MO, USA).

### 3.2. Raw Material

The study was carried out with olive oils obtained from olives of ‘Picual’ variety cultivated as an experimental olive variety (*Olea europaea* L.) located within the limits of the olive-growing ‘Tierra de Barros’ area, in the southwest of Badajoz (Spain). The olive orchard was composed of fifteen-year-old olive trees (plantation frame 6 × 7 m²). The orchard was managed without irrigation, controlling the weeds with post-emergence herbicides. Olives were collected in the morning at the end of October at the veraison stage of maturation, and they were immediately transported to the mill to avoid compositional changes. The oil was extracted within 24 h.

### 3.3. EVOO Samples

**Figure 5:**
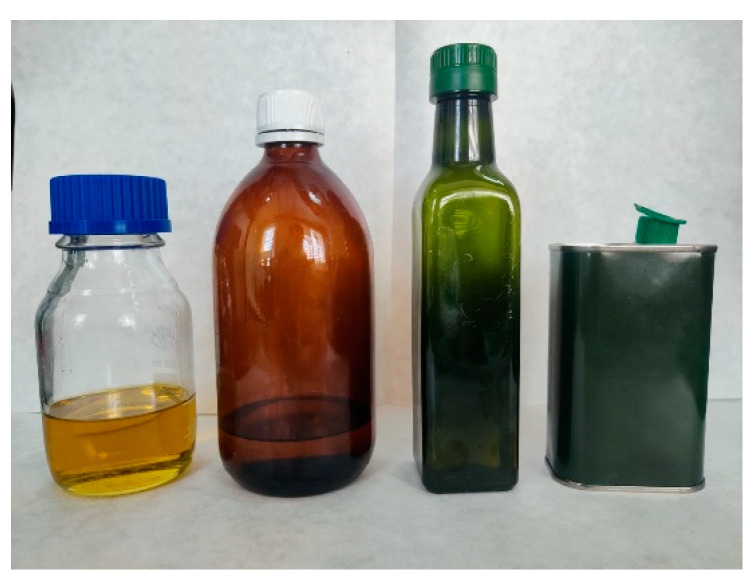
Different containers employed in the study.

The EVOO was obtained from a local industrial oil mill (Pieralisi, Italy) in Badajoz (Spain). Olives were crushed with a hammer mill and malaxation was carried out at 28 °C for 40 min. A two-phase decanter was used to extract the EVOO. The filtration procedure was carried out immediately after oil extraction. The oil obtained was stored in a stainless-steel silo before being packaged for the experimental tests. After 60 days of storage, the EVOO was placed into four different containers of 250 mL with the same headspace in triplicate: (i) transparent crystal (TC); (ii) opaque crystal (OC); (iii) opaque plastic (Pl); and (iv) metallic can (C). The containers were stored at 20 °C in the laboratory for 10 months. Figure 5 shows the different containers.

### 3.4. Determination of Oil Quality Indexes

At the beginning of the storage period and every 2–3 weeks, measurements of the oil samples stored in each container were taken in order to analyze the quality. To determine the general quality index of the virgin olive oils, free fatty acids, peroxide index, and extinction coefficients (K_232_ and K_270_) of EVOO samples were determined following the methods described in Regulations EEC 2568/91 and its subsequent amendments. All parameters were determined in duplicate for all samples.

The free acidity was determined by titration of oil dissolved in ethanol-ethyl ether 96° (1:1 *v*/*v*) with an ethanolic solution of potassium hydroxide 0.1 N, using phenolphthalein as an indicator. The results were expressed as g of oleic acid per 100 g of oil. The peroxide index was determined diluting the oil samples in acetic acid-chloroform, 3:2 *v*/*v*. The dissolved oil was mixed with a solution of acetic acid and potassium iodide in the dark and the released iodine was titrated with a solution of sodium thiosulfate using starch as an indicator. The results were expressed as milliequivalents of active O_2_ per kg of oil. For the analysis of the extinction coefficients, K_270_ and K_232_, the oil was mixed with cyclohexane (1%) and the mixture was introduced in 1 cm optical path quartz cell. After, a UV spectrophotometer (Hewlett-Packard, HP 8452 A) at 270 and 232 nm was used. Finally, the oxidative stability index (OSI) was evaluated in Rancimat 679 equipment (Metrohn Co., Basel, Switzerland). Olive oil (3 g) was placed in a standard glass tube which was heated to 120 °C and had an air flow of 10 L/h. The results were expressed as induction period per hour.

### 3.5. Statistical Analysis

The results were analyzed with variance analysis (ANOVA) and Tukey’s multiple range test. The adequacy of the model was evaluated through a standardized remainder study to check the normality of the data and the homogeneity of the variances. Statistical significance was accepted at a level of *p* < 0.05. For ANOVA, SPSS 18.0 software (SPSS Inc., Chicago, IL, USA) was used. The results are expressed as mean values and the standard deviation (SD) was calculated.

### 3.6. Fluorescence Measurements

Fluorescence excitation emission matrices (EEMs) were obtained on a Cary Eclipse Varian Fluorescence Spectrophotometer, equipped with two Czerny–Turner monochromators, a xenon light source and a photomultiplier tube as detector [17,39]. The Cary Eclipse software 1.2 was used for data acquisition. A 1.0 cm quartz cell was used. Measurements were made with a variable-angle front-face accessory. The angle of incidence, defined as the angle between the excitation beam and the axis perpendicular to the cell surface, was set to 35°. The slits of excitation and emission monochromators were set to 5 nm. The EEMs were registered as a set of emission spectra over a range of excitation wavelengths. The excitation wavelengths ranged from 250 to 450 nm, each with 5 nm increments. At each excitation wavelength, the emission spectra were recorded from 200 to 700 nm, at 2 nm intervals. Each sample was registered twice with two different photomultiplier tube sensitivities: 630 and 700 V. Data were saved in ASCII format and transferred to a PC for the chemometric analysis described in the next section.

### 3.7. Multivariate Analysis

EEM data were exported to ASCII code and processed using Matlab software (Matlab R2016b). The graphical interface MVC2 (http://www.iquirconicet.gov.ar/descargas/mvc2.rar; accessed on 5 May 2021) was used for PARAFAC [32] and U-PLS [40] calculations.

## 4. Conclusions

The present study aimed to investigate the evolution of EVOO stored in different containers at room temperature across 40 weeks, and the use of fluorescence spectroscopy coupled with chemometric algorithms to monitor the samples. Physicochemical analysis showed that olive oil stored in transparent crystal containers suffered the worst deterioration among the samples tested due to the transparency of the containers, followed by opaque crystal containers. In this sense, the peroxide index increased during the first weeks, and the acidity remained constant but increased during the last months of the study, while data regarding the oxidative stability index showed a decrease across the study. This reaffirms that photo-oxidation is much faster than auto-oxidation. In places exposed to light, cans may be the most suitable containers to protect olive oil samples against photo-oxidation, maintaining the EVOO category for as long as possible. In that sense, the use of non-transparent containers should be recommended to keep photosensitive samples intact. The classic analysis together with the use of fluorescence gives us an accurate, complete and sensitive monitoring of the evolution of the components of olive oil in a domestic environment. The advantage of using fluorescence signals as fingerprint of samples is their high selectivity and sensitivity. A three-dimensional map of samples offers a huge amount of information concerning complex samples, as foods are. The different spectral regions obtained with the fluorescence analysis of the oils during storage were associated with degradation processes, overall, with primary and secondary oxidation products. On the other hand, EEMs combined with U-PLS proved to be an excellent tool that allowed the establishment of regression models between quality parameters and EEMs. This proves the accuracy and precision of the developed model for the physico-chemical parameters studied, and suggests that the EEMs combined with U-PLS are a good method to determine these parameters, avoiding the traditional one. For all of that, the combination of the determination of the quality parameters along with analytical techniques such as non-destructive fluorescence results in an interesting methodology for studying olive oil deterioration over time, accelerating the process with minimal cost.

## Figures and Tables

**Table 1:** Statistical parameters with the U-PLS model for the quantification of the quality parameters.

		Peroxide Index	K_232_	K_270_	Oxidative Stability Index (OSI)
Cross-validation	r^2^_CV_	0.91	0.90	0.84	0.92
	RMSECV	1.94 mEqO_2_·kg^−1^	0.22	0.017	3.21 h
	REP (%)	19.07	12.94	14.92	7.51
Validation	r^2^_V_	0.94	0.94	0.86	0.90
	RMSEP	1.53 mEqO_2_·kg^−1^	0.16	0.019	3.60
	REP (%)	14.98	9.22	12.25	8.12

## Data Availability

The authors confirm that the data supporting the findings of this study are available within the article and the raw data that support the findings are available from the corresponding author, upon reasonable request.
